# Sensory experience modulates the reorganization of auditory regions for executive processing

**DOI:** 10.1093/brain/awac205

**Published:** 2022-06-02

**Authors:** Barbara Manini, Valeria Vinogradova, Bencie Woll, Donnie Cameron, Martin Eimer, Velia Cardin

**Affiliations:** Deafness, Cognition and Language Research Centre and Department of Experimental Psychology, UCL, London WC1H 0PD, UK; School of Psychology, University of East Anglia, Norwich NR4 7TJ, UK; Deafness, Cognition and Language Research Centre and Department of Experimental Psychology, UCL, London WC1H 0PD, UK; Norwich Medical School, University of East Anglia, Norwich NR4 7TJ, UK; Department of Psychological Sciences, Birkbeck, University of London, London WC1E 7HX, UK; Deafness, Cognition and Language Research Centre and Department of Experimental Psychology, UCL, London WC1H 0PD, UK

**Keywords:** deafness, executive function, auditory cortex

## Abstract

Crossmodal plasticity refers to the reorganization of sensory cortices in the absence of their typical main sensory input. Understanding this phenomenon provides insights into brain function and its potential for change and enhancement. Using functional MRI, we investigated how early deafness influences crossmodal plasticity and the organization of executive functions in the adult human brain. Deaf (*n* = 25; age: mean = 41.68, range = 19–66, SD = 14.38; 16 female, 9 male) and hearing (*n* = 20; age: mean = 37.50, range = 18–66, SD = 16.85; 15 female, 5 male) participants performed four visual tasks tapping into different components of executive processing: task switching, working memory, planning and inhibition. Our results show that deaf individuals specifically recruit ‘auditory’ regions during task switching. Neural activity in superior temporal regions, most significantly in the right hemisphere, are good predictors of behavioural performance during task switching in the group of deaf individuals, highlighting the functional relevance of the observed cortical reorganization. Our results show executive processing in typically sensory regions, suggesting that the development and ultimate role of brain regions are influenced by perceptual environmental experience.

## Teaser

Using functional MRI, Manini, Vinogradova *et al.* show that the auditory cortex of deaf individuals is involved in task switching. These findings show that sensory cortices can change their function to higher-order cognition, and that sensory experience shapes cortical reorganization and executive processing in the brain.

## Introduction

Sensory systems feed and interact with all aspects of cognition. As such, it is likely that developmental sensory experience will affect the organization of higher-order cognitive processes such as executive functions. Here we studied executive processing in early deaf individuals to understand the influence of early sensory experience on higher-order cognition and neural reorganization.

Executive functions are higher-order cognitive processes responsible for flexible and goal-directed behaviours, which have been associated with activity in frontoparietal areas of the brain.^1^ However, studies on deafness have shown reorganization for visual working memory in regions typically considered to be part of the auditory cortex.^2–5^ These working memory responses in auditory regions suggest that, in the absence of early sensory stimulation, a sensory region can change its function as well as the perceptual modality to which it responds.^6,7^ The adaptation of sensory brain regions to processing information from a different sensory modality is known as crossmodal plasticity.^7–19^ In deaf individuals, crossmodal plasticity often refers to responses to visual or somatosensory stimuli in regions of the superior temporal cortex that in hearing individuals are typically involved in processing sounds.^7–11,14–19^ The common assumption here, and in general when referring to crossmodal plasticity, is that the auditory cortex will preserve its sensory processing function, but process a different type of sensory input. The presence of working memory responses in the auditory regions of deaf individuals takes the concept of crossmodal plasticity further, suggesting that, in the absence of early auditory stimulation, there is a shift from sensory to cognitive processing in such regions. If this is the case, it would suggest that cortical functional specialization for sensory or cognitive processing is partially driven by environmental sensory experience. The aim of our study is to elucidate the role of the auditory cortex of deaf individuals in executive functions, to understand how sensory experience impacts cognitive processing in the brain. Specifically, we tested whether the auditory regions of deaf individuals are involved in cognitive control or whether they have a role in specific subcomponents of executive functions.

To address our aims, we conducted a functional MRI experiment in deaf and hearing individuals. Participants performed tasks tapping into different executive functions: switching, working memory, planning and inhibition. If the auditory cortex of deaf individuals has a role in cognitive control, we would expect all tasks to recruit this region. However, if the auditory areas of deaf individuals are involved in specific subcomponents of executive functioning, these regions will be differentially activated by each of the tasks. If neural activity in the reorganized auditory cortex can predict behavioural performance in deaf individuals, this will corroborate the functional significance of such plasticity effect.^20,21^

## Materials and methods

### Participants

There were two groups of participants (see demographics in Supplementary Tables 1 and 2).

- Twenty-nine congenitally or early (before 3 years of age) severely-to-profoundly deaf individuals whose first language is British Sign Language (BSL) and/or English (Supplementary Table 3). We recruited a larger number of deaf participants to reflect the language variability of the deaf population in the UK, as discussed in the ‘Language assessment’ section. Datasets from three deaf participants were excluded from all analyses due to excessive motion in the scanner. One participant was excluded because they only had a mild hearing loss in their best ear (pure-tone average <25 dB). In total, 25 deaf participants were included in the analysis of at least one executive function task (see Supplementary Table 4 for details on exclusion).
- Twenty hearing individuals who are native speakers of English with no knowledge of any sign language.

Deaf and hearing participants were matched on age, gender, reasoning and visuospatial working memory span (Supplementary Table 2).

All participants gave written informed consent. All procedures followed the standards set by the Declaration of Helsinki and were approved by the ethics committee of the School of Psychology at the University of East Anglia and the Norfolk and Norwich University Hospital Research and Development department.

Participants were recruited through public events, social media and participant databases of the University College London Deafness, Cognition and Language Research Centre and the University of East Anglia School of Psychology. Participants were all right-handed (self-reported), had full or corrected vision and no history of neurological conditions. All participants were compensated for their time, travel and accommodation expenses.

### General procedure

Participants took part in one behavioural and one scanning session. The sessions took place on the same or different days.

The behavioural session included:

- Standardized reasoning and working memory tests: the Block Design subtest of the Wechsler Abbreviated Scale of Intelligence^22^ and the Corsi Block-tapping test^23^ implemented in psychology experiment building language software^24^ (PEBL, http://pebl.sourceforge.net/).
- Language tasks: four tasks were administered to assess language proficiency in English and BSL in deaf participants (see the ‘Language assessment’ section).
- Pre-scanning training: the training session ensured that participants understood the tasks and reached accuracy of at least 75%. The tasks were explained in the participant’s preferred language (English or BSL). A written description of all the tasks was provided to all participants (deaf and hearing) to support the experimenter’s explanation.
- Audiogram screening: pure-tone averages were used to measure the degree of deafness in deaf participants. Copies of audiograms were provided by the participants from their audiology clinics or were collected at the time of testing using a Resonance R17 screening portable audiometer. Participants included in the study had a mean pure-tone average >75 dB averaged across the speech frequency range (0.5, 1, 2 kHz) in both ears (mean = 93.66 ± 7.79 dB; range: 78.33–102.5 dB). Four participants did not provide their audiograms, but they were all congenitally severely or profoundly deaf and communicated with the researchers using BSL or relying on lipreading.

During the scanning session, functional MRI data were acquired while participants performed four visual executive function tasks on switching, working memory, planning and inhibition (see details next). The order of the tasks was counterbalanced across participants.

### Experimental design

**Figure 1:**
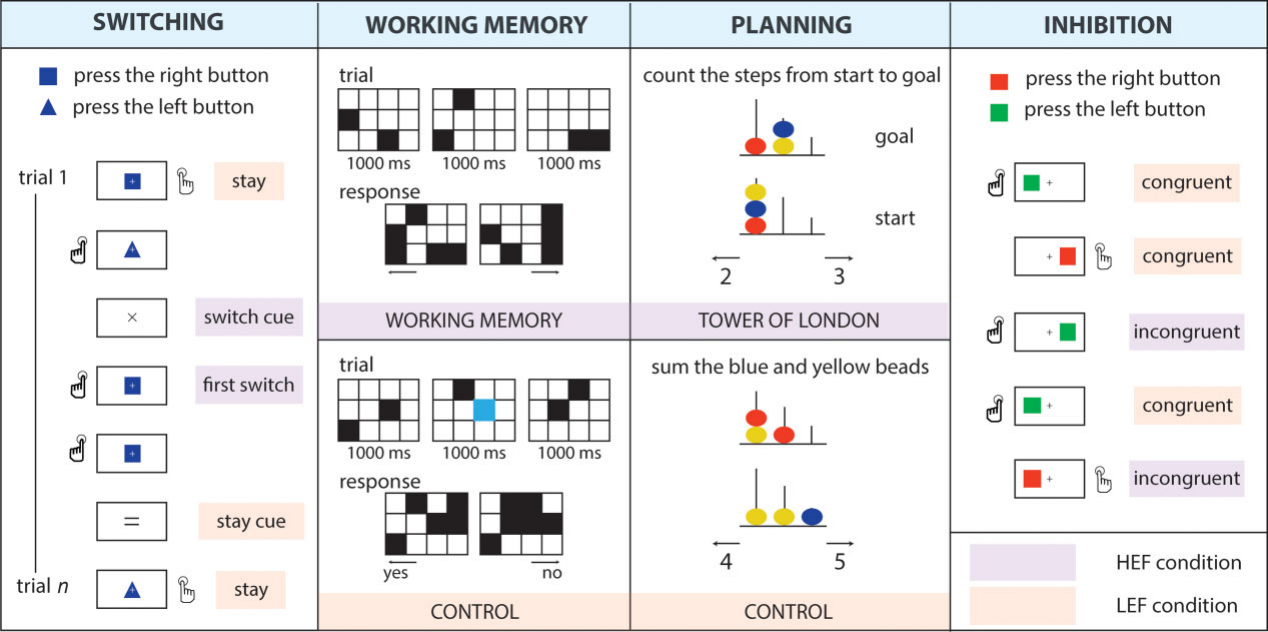
**Executive function tasks.** Each task had a higher executive demands condition (HEF, purple) and a lower executive demands condition (LEF, peach). See the ‘Materials and methods’ section for details of the design.

All tasks were designed so that each had one condition with higher executive demands (higher executive function, HEF) and one with lower demands (lower executive function; LEF) (Fig. 1).

For the switching task, participants had to respond to the shape of geometric objects, i.e. a rectangle and a triangle^25,26^ (Fig. 1). At the beginning of the run, participants were instructed to press a key with their left hand when they saw a rectangle and with their right hand when they saw a triangle. Each block started with a cue indicating that the task was to either keep the rule they used in the previous block (‘stay’ trials; LEF) or to switch it (‘switch’ trials; HEF). In the switch trials, participants had to apply the opposite mapping between the shape and the response hand. Each block included the presentation of the instruction cue (200 ms), a fixation cross (500 ms) and two to five task trials. During each trial, a geometrical shape (either a blue rectangle or a blue triangle) was shown at the centre of the screen until the participant responded for a max of 1500 ms. Visual feedback (500 ms) followed the participant’s response. There were 230 trials in 80 blocks of either the LEF (40) or HEF (40) condition. The analysis for the HEF condition only included the first trial of the switch block (see ‘Statistical analysis of behavioural performance’ section).

For the working memory task, we used a visuospatial task^27,28^ contrasted with a perceptual control task (Fig. 1). A visual cue (1500 ms) indicated which task participants should perform. The cue was followed by a 3 × 4 grid. Black squares were displayed two at a time at random locations on the grid for 1000 ms, three times. In the HEF condition, participants were asked to memorize the six locations. Then they indicated their cumulative memory for these locations by choosing between two grids in a two-alternative, forced-choice paradigm via a button press. The response grids were displayed until the participant responded, or for a maximum of 3750 ms. In the control condition (LEF), participants indicated whether a blue square was present in any of the grids, ignoring the configuration of the highlighted squares. Trials were separated by an inter-trial interval with duration jittered between 2000 and 3500 ms. Each experimental run had 30 working memory trials and 30 control trials.

For the planning task, we used a computer version of the classic Tower of London task^29,30^ (Fig. 1). In each trial, two configurations of coloured beads placed on three vertical rods appeared on a grey screen, with the tallest rod containing up to three beads, the middle rod containing up to two beads and the shortest rod containing up to one bead. In the Tower of London condition (HEF), participants had to determine the minimum number of moves needed to transform the starting configuration into the goal configuration following two rules: (i) only one bead can be moved at a time; and (ii) a bead cannot be moved when another bead is on top. There were four levels of complexity, depending on the number of moves required (2, 3, 4 and 5). In the control condition (LEF), participants were asked to count the number of yellow and blue beads in both displays. For both conditions, two numbers were displayed at the bottom of the screen: one was the correct response and the other was incorrect by +1 or −1. Participants answered with their left hand when they chose the number on the left side of the screen, and with their right hand when their choice was on the right. The maximum display time for each stimulus was 30 s. The duration of the inter-trial interval was jittered between 2000 and 3500 ms. There were 30 trials in the Tower of London condition and 30 trials in the control condition.

To study inhibitory control, we used Kelly and Milham’s^31^ version of the classic Simon task (https://exhibits.stanford.edu/data/catalog/zs514nn4996). A square appeared on the left or the right side of the fixation cross. The colour of the squares was the relevant aspect of the stimuli, with their position irrelevant for the task. Participants were instructed to respond to the red square with the left hand and the green square with the right hand. In the congruent condition (LEF), the button press response was spatially congruent with the location of the stimuli (e.g. the right-hand response for a square appearing on the right side of the screen) (Fig. 1). In the incongruent condition (HEF), the correct answer was in the opposite location in respect to the stimulus. Half of the trials were congruent, and half were incongruent. Each stimulus was displayed for 700 ms, with a response window of up to 1500 ms. The inter-trial interval was 2500 ms for most trials, with additional blank intervals of 7.5 s (20), 12.5 s (2) and 30 s (1). Participants completed one or two runs of this task, each consisting of a maximum of 200 trials.

### Statistical analysis of behavioural performance

Averaged accuracy (%correct) and reaction time were calculated. For each participants’ set of reaction times, we excluded outlier values where participants responded too quickly or where they took a long time to respond. We did this by calculating each participant’s interquartile range separately, and then removing values that were >1.5 interquartile ranges below the first quartile or above the third quartile of the data series. Differences between groups on accuracy or reaction time were investigated with repeated-measures ANOVAs with between-subjects factor group (hearing, deaf) and within-subjects factor condition (LEF, HEF).

In the switching task, the accuracy switch cost (SwitchCost_ACC_) was calculated as the difference in the percentage of errors (%errors) between the first switch trial of a switch block and all stay trials. reaction time switch cost (SwitchCost_RT_) was calculated as the difference in reaction time between the first switch trial of a switch block and all stay trials.

In the inhibition task, the Simon effect was calculated as the difference in %errors or reaction time between the incongruent and congruent trials.

### Image acquisition

Images were acquired at the Norfolk and Norwich University Hospital in Norwich, UK, using a 3 T wide bore GE 750W MRI scanner and a 64-channel head coil. Communication with the deaf participants occurred in BSL through a close-circuit camera, or through written English through the screen. An intercom was used for communication with hearing participants. All volunteers were given ear protectors. Stimuli were presented with PsychoPy software^32^ (https://psychopy.org) through a laptop (MacBook Pro, Retina, 15-inch, Mid 2015). All stimuli were projected by an AVOTEC’s Silent Vision projector (https://www.avotecinc.com/high-resolution-projector) onto a screen located at the back of the magnet’s bore. Participants watched the screen through a mirror mounted on the head coil. Button responses were recorded via fibre-optic response pad button boxes (https://www.crsltd.com/tools-for-functional-imaging/mr-safe-response-devices/forp/). Functional imaging data were acquired using a gradient-recalled echo planar imaging sequence [50 slices, repetition time (TR) = 3000 ms, echo time (TE) = 50 ms, field of view (FOV) = 192 × 192 mm, 2 mm slice thickness, distance factor 50%] with an in-plane resolution of 3 × 3 mm. The protocol included six functional scans: five task-based functional MRI scans (switching: 10.5 min, 210 volumes; working memory: 11 min, 220 volumes; planning: 11.5 min, 230 volumes; inhibition: two runs of 10 min, 200 volumes each) and one resting state scan (part of a different project, and to be reported in a different paper). Some participants did not complete all functional scans (Supplementary Table 4). An anatomical T_1_-weighted scan [IR-FSPGR, inversion time = 400 ms, 1 mm slice thickness] with an in-plane resolution of 1 × 1 mm was acquired during the session.

Raw B0 field map data were acquired using a 2D multi-echo gradient-recalled echo sequence with the following parameters: TR = 700 ms, TE = 4.4 and 6.9 ms, flip angle = 50°, matrix size = 128 × 128, FOV = 240 mm × 240 mm, number of slices = 59, thickness = 2.5 mm and gap = 2.5 mm. Real and imaginary images were reconstructed for each TE to permit calculation of B0 field maps in Hz.^33–35^

### Functional MRI preprocessing

Functional MRI data were analysed with MATLAB 2018a (MathWorks, MA, USA) and Statistical Parametric Mapping software (SPM12; Wellcome Trust Centre for Neuroimaging, London, UK).^36^ The anatomical scans were segmented into different tissue classes: grey matter, white matter and CSF. Skull-stripped anatomical images were created by combining the segmented images using the Image Calculation function in SPM (ImCalc, http://tools.robjellis.net). The expression used was: {[i1.*(i2 + i3 + i4)] > threshold}, where i1 was the bias-corrected anatomical scan and i2, i3 and i4 were the tissue images (grey matter, white matter and CSF, respectively). The threshold was adjusted between 0.5 and 0.9 to achieve adequate brain extraction for each participant. Each participant’s skull-stripped image was normalized to the standard MNI space (Montreal Neurological Institute) and the deformation field obtained during this step was used for normalization of the functional scans. Susceptibility distortions in the echo-planar images were estimated using a field map that was coregistered to the blood oxygenation level-dependent (BOLD) reference.^33,34^ Images were realigned using the precalculated phase map, coregistered, slice-time corrected, normalized and smoothed (using an 8-mm full-width at half-maximum Gaussian kernel). All functional scans were checked for motion and artefacts using the ART toolbox (https://www.nitrc.org/projects/artifact_detect).

### Functional MRI first-level analysis

The first-level analysis was conducted by fitting a general linear model with regressors of interest for each task (see details next). All the events were modelled as a boxcar and convolved with SPM’s canonical haemodynamic response function. The motion parameters, derived from the realignment of the images, were added as regressors of no interest. Regressors were entered into a multiple regression analysis to generate parameter estimates for each regressor at every voxel.

For the switching task, the first trial of each switch block (HEF) and all stay trials (LEF) were modelled as regressors of interest separately for the left- and right-hand responses. The cues and the remaining switch trials were included as regressors of no interest.

For the working memory task, the conditions of interest were working memory (HEF) and control (LEF). The onset was set at the presentation of the first grid, with the duration set at 3.5 s (i.e. the duration of the three grids plus a 500 ms blank screen before the appearance of the response screen; Fig. 1). Button responses were included separately for each hand and condition as regressors of no interest.

For the planning task, the Tower of London (HEF) and control (LEF) conditions were included in the model as regressors of interest, with onsets at the beginning of each trial and duration set to the trial-specific reaction time. Button responses were modelled separately for each hand as regressors of no interest.

For the inhibition task, four regressors of interest were obtained by combining the visual hemifield where the stimulus appeared with the response hand [(i) right visual hemifield*—*left hand; (ii) left visual hemifield*—*right hand; (iii) right visual hemifield*—*right hand; (iv) left visual hemifield*—*left hand]. Right visual hemifield*—*left hand and left visual hemifield*—*right hand were the incongruent conditions (HEF), whereas the right visual hemifield-right hand and left visual hemifield-left hand were the congruent conditions (LEF).

### Region of interest analysis

**Figure 2:**
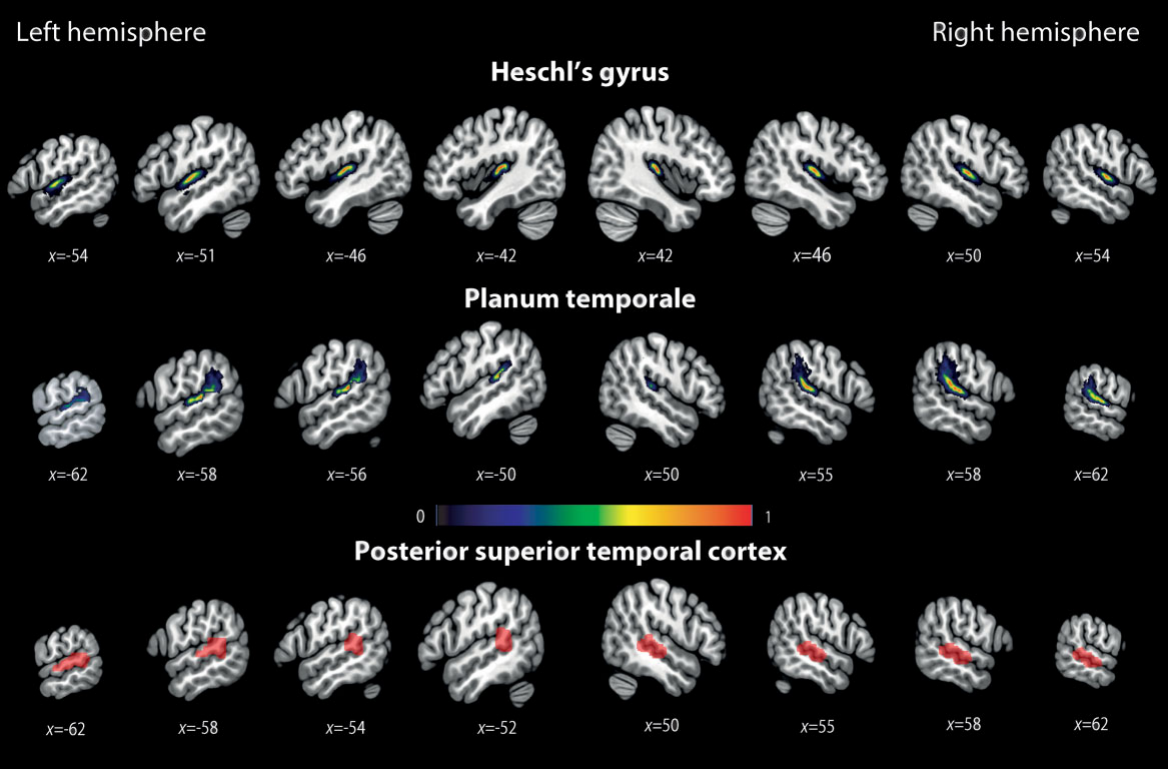
**Temporal ROIs.** Temporal regions included in the analysis: HG, PT and superior temporal cortex (pSTC). HG and PT were defined anatomically, in a subject-specific manner, using the FreeSurfer software package.^36^ The figure shows the overlap of all subject-specific ROIs. Common voxels between left PT and left pSTC have been subtracted from left PT (see ‘Materials and methods’ section). The pSTC was defined functionally, based on the findings of Cardin *et al*.^3^ (see the ‘Materials and methods’ section).

We conducted a region of interest (ROI) analysis to investigate crossmodal plasticity and differences between groups in the auditory cortex. Three auditory regions of the superior temporal cortex were included in this analysis: Heschl’s gyrus (HG), the planum temporale (PT) and the posterior superior temporal cortex (pSTC) (Fig. 2). HG and the PT were defined anatomically, using FreeSurfer software^37^ (https://surger.nmr.mgh.harvard.edu). Full descriptions of these procedures can be found elsewhere,^38,39^ but, in short, each participant’s bias-corrected anatomical scan was parcellated and segmented, and voxels with the HG label and the PT label were exported using SPM’s ImCalc function (http://robjellis.net/tools/imcalc_documentation.pdf). Participant-specific ROIs were then normalized to the standard MNI space using the deformation field from the normalization step of the preprocessing.

The pSTC was specified following findings from Cardin *et al*.^4^ where a visual working memory crossmodal plasticity effect was found in right and left pSTC in deaf individuals [left: −59, −37, 10; right: 56, −28, −1]. Right and left functional pSTC ROIs were defined using data from Cardin *et al*.,^3^ with the contrast [deaf (working memory > control task) > hearing (working memory > control task)] (*P* < 0.005, uncorrected).

There was an average partial overlap of 8.2 voxels (SD = 6.86) between left PT and left pSTC, with no significant difference in overlap between groups (deaf: mean = 9.92, SD = 7.02; hearing: mean = 6.05, SD = 6.17). To ensure that the two ROIs were independent, common voxels were removed from left PT in a subject-specific manner. Removing the overlapping voxels did not qualitatively change the results.

Parameter estimates for each participant were extracted from each ROI using MarsBaR v.0.44^40^ (http://marsbar.sourceforge.net). The data were analysed using JASP^41^ (https://jasp-stats.org) and entered into separate mixed repeated measures ANOVAs for each task and set of ROIs. Factors in the ANOVAs on the temporal ROIs included: the between-subjects factor group (hearing, deaf) and the within-subjects factors ROI (HG, PT, pSTC), hemisphere (left, right) and condition (LEF, HEF).

The Greenhouse–Geisser correction was applied when the assumption of sphericity was violated. Significant interactions and effects of interest were explored with Student’s *t*-tests or Mann–Whitney U-tests when the equal variance assumption was violated.

### Language assessment

We recruited a representative group of the British deaf population, who usually have different levels of proficiency in sign and spoken language. This was: (i) to study plasticity in a representative group of deaf individuals; and (ii) to study the relationship between language experience and the organization of cognitive networks of the brain, which will be reported in a separate paper.

To assess the language proficiency of deaf participants, we chose grammaticality judgement tests measuring language skills in English and BSL. The BSL grammaticality judgement task (BSLGJT) is described in Cormier *et al*.^42^ and the English grammaticality judgement task (EGJT) was designed based on examples from Linebarger *et al*.^43^ The BSLGJT and the EGJT use a single method of assessing grammaticality judgements of different syntactic structures in English and BSL. Grammaticality judgement tests have been used in deaf participants before and have proved to be efficient in detecting differences in language proficiency among participants with varying ages of acquisition.^42,44^ Deaf participants performed both the BSL and English tests if they knew both languages, or only the English tests if they did not know BSL.

To control for potential language proficiency effects, we combined results from the EGJT and BSLGJT to create a single, modality-independent measure of language proficiency in the deaf group. Accuracy scores in the EGJT (%correct; mean = 83.51, SD = 11.4, *n* = 25) and BSLGJT (mean = 77.88, SD = 13.1, *n* = 21) were transformed into *z*-scores separately for each test. For each participant, the EGJT and BSLGJT *z*-scores were then compared, and the higher one was chosen for a combined modality-independent language proficiency score (Supplementary Fig. 1).

### Multiple linear regression

Multiple linear regression analyses were conducted to investigate whether neural activity in the superior temporal cortex of deaf individuals can predict performance in the switching task. The data were analysed using a backward data entry method in JASP.^41^ The default stepping method criteria were used, where predictors with *P* < 0.05 are entered into the model and those with *P* > 0.1 are removed until all predictors fall within these criteria. SwitchCost_RT_ and SwitchCost_ACC_ were entered as dependent variables in separate analyses. Each regression analysis had three covariates: neural switch cost in the right hemisphere, neural switch cost in the left hemisphere and language.

Neural switch cost (BOLD_switch_ − BOLD_stay_) was calculated in ROIs with significant differences between the switch and stay condition in the deaf group. The average neural activity in all stay trials (BOLD_stay_) was subtracted from the average activity in the first switch trials (BOLD_switch_), and then averaged across ROIs separately in the right and left hemisphere.

### Data availability

The data and analysis files for this paper can be found at https://osf.io/uh2ap/.

## Results

### Behavioural results

**Figure 3:**
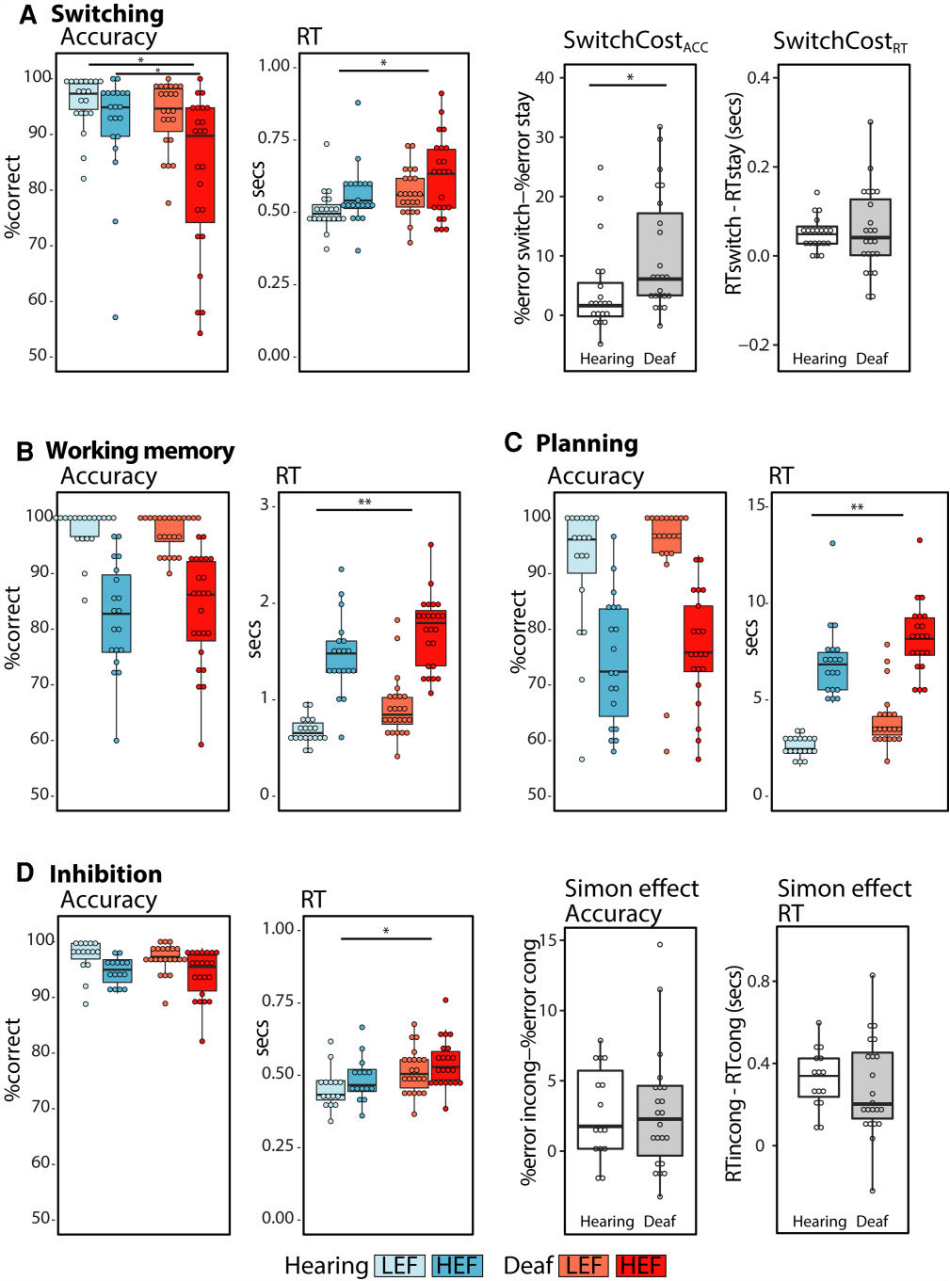
**Behavioural performance.** The figure shows average accuracy (%correct) and reaction time (RT, in seconds) for each task and condition in the hearing and the deaf groups. It also shows the average switch costs and Simon effects for both accuracy and reaction time in each group. The SwitchCost_ACC_ and Simon effect are calculated and plotted using %error instead of %correct, so that larger values indicate an increase in cost. Only the first trials of the switch blocks were included in the HEF condition. The bold lines in the box plots indicate the median. The lower and upper hinges correspond to the first and third quartiles. Statistically significant (*P* < 0.05) differences between conditions are not shown in the figure, but were found for all tasks in both groups (Supplementary Table 5). **P < 0.01; *P < 0.05.

Deaf (*n* = 25) and hearing (*n* = 20) individuals were scanned while performing four executive function tasks: switching, working memory, planning and inhibition (Fig. 1). Behavioural results from all tasks are shown in Fig. 3. To explore differences in performance between groups, we conducted 2 × 2 repeated-measures ANOVAs for each task, with either accuracy or reaction time as the dependent variable, between-subjects factor group (hearing, deaf) and within-subjects factor condition (HEF, LEF). Results show a significant main effect of condition for both accuracy and reaction time in all tasks, confirming that the HEF condition was more difficult and demanding than the LEF condition (Supplementary Table 5).

The group of deaf individuals had significantly slower reaction times in all tasks (Supplementary Table 5). Switching was the only task where there was a significant main effect of group on accuracy [*F*(1,41) = 4.32, *P* = 0.04, η^2^_p_ = 0.09], as well as a Condition × Group interaction [*F*(1,41) = 4.98, *P* = 0.03, η^2^_p_ = 0.11]. A *post hoc t*-test revealed a significant between-groups difference, where the group of deaf individuals was significantly less accurate than the group of hearing individuals in the switch condition [*t*(41) = −2.22, *P* = 0.03, Cohen's *d* = 0.68]. The difference in SwitchCost_ACC_ (%errors_switch_ − %errors_stay_) reflects the significant interaction, with the deaf group [mean = 10.24, SD = 9.89, *t*(22) = 4.96, *P* < 0.001, *d* = 1.03] having a larger SwitchCost_ACC_ than the hearing group [mean = 4.18; SD = 7.53, *t*(19) = 2.49, *P* = 0.02, *d* = 0.56; Fig. 3A].

### Functional MRI results

Functional MRI results show that all executive function tasks activated typical frontoparietal regions in both groups of participants (Supplementary Fig. 2). There were significantly stronger activations in the HEF condition in the switching, working memory and planning tasks. These included commonly found activations in frontoparietal areas, such as dorsolateral prefrontal cortex, frontal eye fields, presupplementary motor area and intraparietal sulcus. In the inhibition task, the HEF incongruent condition resulted in stronger activation in intraparietal sulcus and left frontal eye fields, but there were no significant differences between conditions.

**Figure 4:**
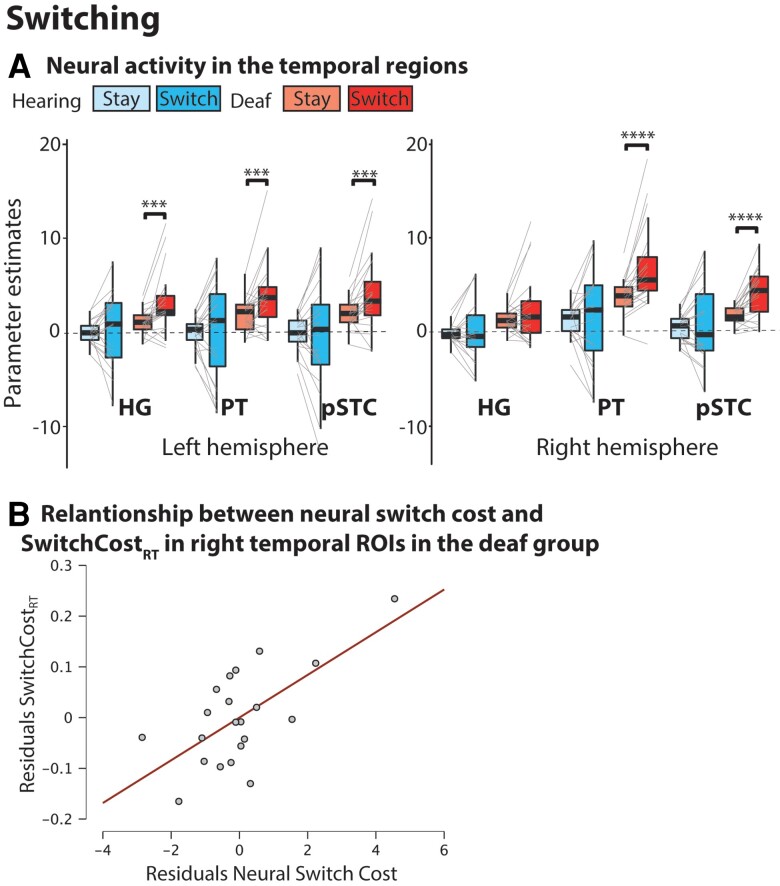
**Switching task analysis.** (**A**) Neural activity in temporal ROIs. ***P < 0.005; ****P < 0.001. (**B**) Partial correlation plot between SwitchCost_RT_ and neural switch cost in right temporal ROIs in the group of deaf individuals. Partial correlation from a multiple linear model with SwitchCost_RT_ as the dependent variable and the following covariates: right hemisphere neural switch cost, left hemisphere neural switch cost, and language.

To investigate crossmodal plasticity and executive processing in the auditory cortex of deaf individuals, we conducted a ROI analysis on superior temporal auditory ROIs. These included: HG, the PT and the pSTC (Fig. 2). Differences and interactions between groups are discussed next, and we first present results from the switching task where we observed the strongest activations of temporal ROIs in the deaf group (Fig. 4). Results from all other tasks are discussed in the following subsection.

### Task switching activates auditory areas in deaf individuals and this activation predicts behaviour

Of the four tasks that we tested, only in the switching task we found both a significant main effect of group [*F*(1,41) = 15.48, *P* < 0.001, η^2^_p_ = 0.27] and a significant interaction between Group × Condition [*F*(1,41) = 4.75, *P* = 0.03, η^2^_p_ = 0.10] (Table 1). The interaction was driven by a significant difference between conditions in the deaf group, but not in the hearing group [deaf_HEFvLEF_: *t*(22) = 4.06, *P* = <0.001, *d* = 0.85; hearing_HEFvLEF_: *t*(19) = 0.26, *P* = 0.79, *d* = 0.06]. To test whether differences between conditions were significant between the switch and stay condition in all ROIs, we conducted *post hoc t*-tests in each ROI and group. This accounted for a total of 12 separate *t*-tests, and to correct for multiple comparisons, we only considered significant those results with *P* < 0.004 (*P* < 0.05/12 = 0.004; corrected *P* < 0.05). We found significant differences between the switch and stay condition in all the left hemisphere ROIs and in the right PT and right pSTC in the deaf group (Fig. 4 and Supplementary Table 6).

**Table 1:** Group main effects and group interactions for all tasks in the ROI analysis

	Switching		Working memory		Planning		Inhibition	
	*F* (d.f.)	*P*	*F* (d.f.)	*P*	*F* (d.f.)	*P*	*F* (d.f.)	*P*
Group	**15.48** (**1,41)**	**<0**.**001**	0.04 (1,41)	0.85	**5.85** (**1,38)**	**0**.**02**	0.03 (1,35)	0.87
Condition × Group	**4.75** (**1,41)**	**0**.**03**	**6.40** (**1,41)**	**0**.**01**	0.56 (1,38)	0.46	0.18 (1,35)	0.67
ROI × Group	**3.42** (**1.9,79.1)**	**0**.**04**	1.18 (1.7,68.4)	0.30	0.73 (1.7,64.6)	0.46	**3.92** (**1.9,66.1)**	**0**.**03**
Hemisphere × Group	0.009 (1,41)	0.92	0.01 (1,41)	0.93	0.46 (1,38)	0.50	0.30 (1,35)	0.59

Significant results are indicated in bold. Full results for each ANOVA can be found in OSF: https://osf.io/dt827/.

To investigate the behavioural relevance of the observed crossmodal plasticity, we evaluated whether neural activity in the superior temporal cortex of deaf individuals can predict performance during the switching task. We conducted two separate multiple linear regression analyses, one with SwitchCost_RT_ and one with SwitchCost_ACC_ as dependent variables (Table 2). The covariates included in the model were: right hemisphere neural switch cost, left hemisphere neural switch cost and language *z*-scores. For the neural swich cost covariates, data was averaged from ROIs in the right and left hemisphere to reduce the number of dimensions in the multiple linear regression models. To do this, we calculated the neural switch cost (BOLD_switch_ − BOLD_stay_) for each ROI with significant differences in activity between the switch and stay conditions in the deaf group (Fig. 4 and Supplementary Table 6), and we then averaged neural switch cost separately for ROIs in the right and left hemisphere. We also included language as a covariate in our models because language proficiency has been shown to modulate performance in executive function tasks in deaf individuals.^45–48^

**Table 2:** Multiple linear regression predicting behavioural performance in the switching task

**SwitchCost_RT_**						
**Model summary**						
**Model**	***R* ^2^**	**Adjusted *R*^2^**	***F***	***P***		
1	**0.46**	**0**.**36**	**4**.**78**	**0**.**01**		
2	**0.41**	**0**.**34**	**6**.**15**	**0**.**009**		
**Coefficients**						
**Model**		**Unstandardized**	**SE**	**Standardized**	***t***	***P***
1	(Intercept)	0.03	0.04		0.63	0.53
	Language score	−0.06	0.05	−0.23	−1.27	0.22
	**LH neural switch cost**	**−0**.**02**	**0**.**01**	**−0**.**69**	**−2**.**35**	**0**.**03**
	**RH neural switch cost**	**0**.**04**	**0**.**01**	**1**.**05**	**3**.**60**	**0**.**002**
2	(Intercept)	−0.01	0.03		−0.51	0.62
	LH neural switch cost	−0.02	0.01	−0.61	−2.09	0.05
	**RH neural switch cost**	**0**.**04**	**0**.**01**	**0**.**99**	**3**.**39**	**0**.**003**

**SwitchCost_ACC_**						
**Model summary**						
**Model**	***R*²**	**Adjusted *R*²**	***F***	***P***		
1	0.28	0.16	2.26	0.12		
**2**	**0.28**	**0**.**20**	**3**.**55**	**0**.**05**		
**3**	**0.21**	**0**.**16**	**4**.**96**	**0**.**04**		
**Coefficients**						
**Model**		**Unstandardized**	**SE**	**Standardized**	***t***	***P***
1	(Intercept)	15.84	4.82		3.28	0.004
	**Language score**	**−12**.**85**	**5**.**83**	**−0**.**46**	**−2**.**20**	**0**.**04**
	LH neural switch cost	−0.28	1.25	−0.08	−0.22	0.82
	RH neural switch cost	1.41	1.41	0.33	1.00	0.33
2	(Intercept)	15.78	4.69		3.37	0.003
	**Language score**	**−12**.**57**	**5**.**55**	**−0**.**45**	**−2**.**27**	**0**.**04**
	RH neural switch cost	1.16	0.84	0.27	1.38	0.18
3	(Intercept)	18.90	4.20		4.50	<0.001
	**Language score**	**−12**.**64**	**5**.**68**	**−0**.**45**	**−2**.**23**	**0**.**04**

Significant results are indicated in bold. LH = left hemisphere; RH = right hemisphere; SE = standard error.

Results from the multiple linear regression analysis using backward data entry show that neural activity in temporal ROIs can significantly predict SwitchCost_RT_ in the deaf group (Table 2). The most significant model included both right and left hemisphere neural switch cost as covariates, and explained 40.6% of the variance [*F*(2,18) = 6.15, *P* = 0.009, *R*^2^ = 0.41, adjusted *R*^2^ = 0.34; Table 2, top section]. There was a positive association between SwitchCost_RT_ and neural switch cost in right hemisphere temporal areas (B = 0.04, SE = 0.01, *β* = 0.99; *P* = 0.003). This means that for every unit increase in neural switch cost in right temporal areas, there is an increase of 40 ms in SwitchCost_RT_. In standardized terms, as neural switch cost increases by 1 SD, SwitchCost_RT_ increases by 0.99 SDs. On the other hand, there was a negative association between the left hemisphere neural and SwitchCost_RT_. However, this was only significant in the full model (*P* = 0.031, B = −0.02, SE = 0.01, *β* = −0.69), but not in the best model (*P* = 0.05, B = −0.02, SE = 0.01, *β* = −0.61; Table 2). There was no significant association between SwitchCost_RT_ and language (B = −0.06, SE = 0.05, *β* = −0.23; *P* = 0.22).

When evaluating whether neural switch cost could also predict SwitchCost_ACC_, we found no significant association between these variables (Table 2, bottom section). Instead, the most significant model included only language as a regressor (Table 2), explaining 20.7% of the variance [*F*(1,19) = 4.96, *P* = 0.04, *R*^2^ = 0.21, adjusted *R*^2^ = 0.16]. For every unit increase in language *z*-scores, there is a decrease of 12.6 units in SwitchCost_ACC_. In standardized terms, as language *z*-scores increased by 1 SD, SwitchCost_ACC_ decreased by 0.45 SDs.

### Recruitment of auditory areas in deaf individuals is not ubiquitous across executive function tasks

**Figure 5:**
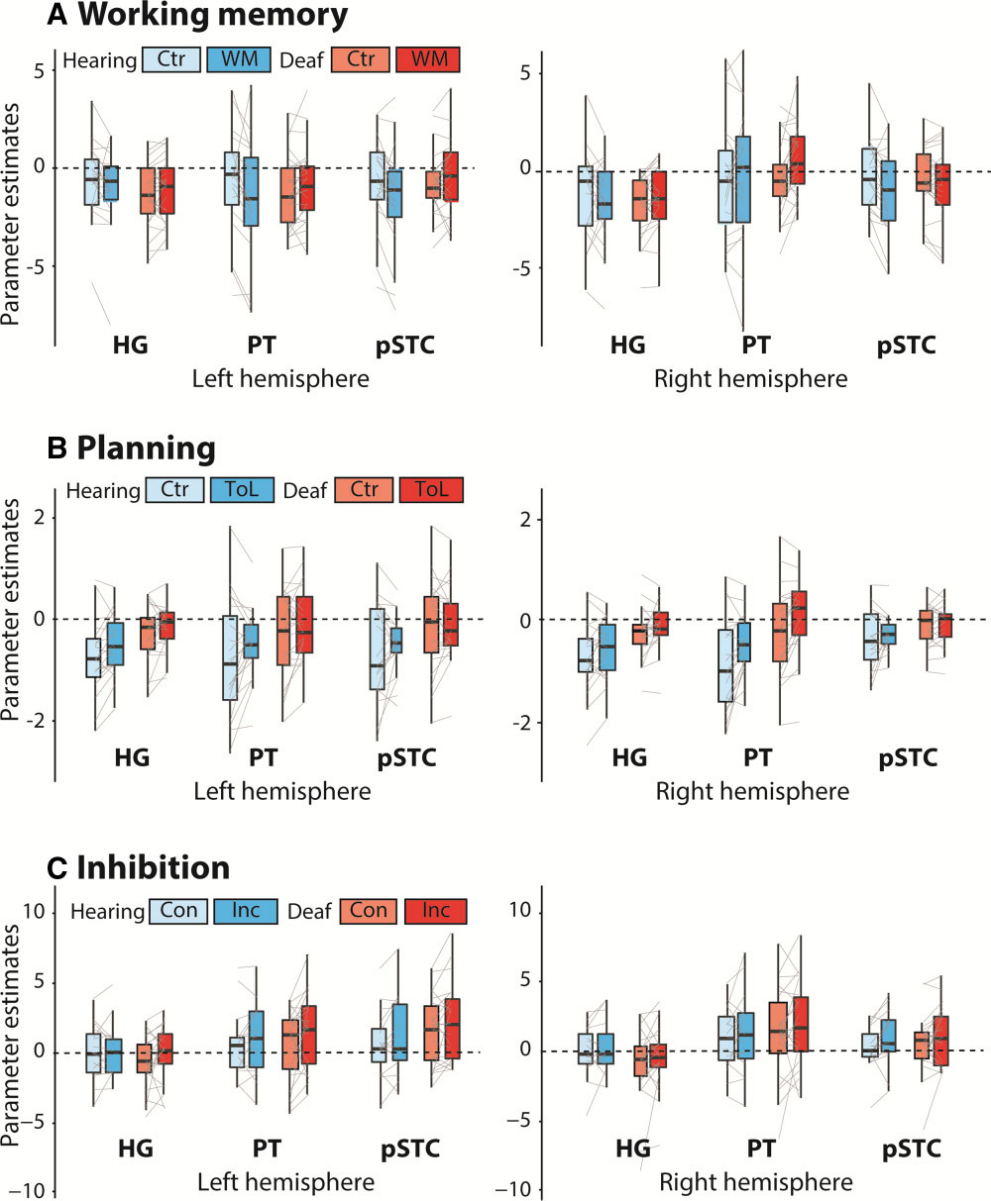
**ROI results from the working memory (A), planning (B) and inhibition (C) tasks.** Ctr = control; WM = working memory; ToL = Tower of London; Con = congruent; Inc = incongruent.

Results from the working memory, planning and inhibition tasks are shown in Fig. 5. In the working memory task, there was a significant Condition × Group interaction [Table 1, *F*(1,41) = 6.41, *P* = 0.01, η^2^_p_ = 0.13], but differences between conditions within each group were not significant [hearing_HEFvLEF_: *t*(18) = −1.74, *P* = 0.10, *d* =−0.40; deaf_HEFvLEF_: *t*(23) = 1.81, *P* = 0.08, *d* = 0.37]. In the planning task, there was a significant main effect of group [*F*(1,38) = 5.85, *P* = 0.02, η^2^_p_ = 0.13], but this was driven by significant deactivations in the hearing group [*t*(18) = −4.47, *P* < 0.001, *d* = −1.00], with no significant difference in activity from baseline in the deaf group [*t*(20) = −1.31, *P* = 0.21, *d* = −0.29]. In the inhibition task, there was a significant interaction between ROI and Group [*F*(1.89,66.05 = 3.92, *P* = 0.03, η^2^_p_ = 0.10]. However, there were no significant differences between groups in any ROI (https://osf.io/9fuec). Instead, the ROI × Group interaction was driven by a main effect of ROI in the deaf group (higher activations for PT and pSTC than HG, https://osf.io/2z35e/), which was not present in the hearing group (https://osf.io/gmy6v/).

## Discussion

We investigated how early sensory experience impacts the organization of executive processing in the brain. We found that, in deaf individuals, primary and secondary auditory areas are recruited during a visual switching task. These results indicate that the sensory or cognitive specialization of cortical regions in the adult brain can be influenced by developmental sensory experience. It is possible that an early absence of auditory inputs results in a shift of functions in regions typically involved in auditory processing, with these regions then adopting a role in specific components of executive processing. Neural activity in temporal regions during the switching task predicted performance in deaf individuals, highlighting the behavioural relevance of this functional shift.

Our design allowed us to thoroughly examine the role of auditory regions in different executive function tasks and determine whether these regions are involved in cognitive control. Previous studies have suggested an involvement of auditory cortex during higher-order cognitive tasks in deaf individuals,^4,5^ but given the focus on a single task, with an experimental and control condition, they cannot inform whether plasticity effects are specific to the demands of the task. Our design included four different visuospatial executive function tasks, all with an experimental (HEF) and control (LEF) condition, probing a variety of executive processes. We found that the HEF condition in all tasks recruited frontoparietal areas typically involved in executive functioning and cognitive control. However, only switching resulted in significant activations in temporal auditory regions in the deaf group. This finding demonstrates that the auditory cortex of deaf individuals serves a specific subcomponent of executive functioning during switching, and not a shared computation across tasks, such as cognitive control. This was not only found in higher-order auditory areas, but also in the left HG, showing that a functional shift towards cognition can indeed occur in primary sensory regions. A significant activation during the switching condition in the left, but not the right HG, provides further evidence for different roles of left and right temporal regions in deaf individuals (see Cardin *et al*.^7^ for a review). Differences in the recruitment of the left and right HG in this study may be linked to the specialization of these regions for sound processing in hearing individuals. In this group, left HG is specialized for the temporal processing of auditory signals, whereas the right HG shows stronger sensitivity to spectral components.^49^ The switching task in this study requires tracking a sequence of stimuli in time, while the extraction of spectral or frequency information is not needed in this task, which could explain the different recruitment of HG across hemispheres. The fact that the right HG was not recruited during the switching task, while right PT and pSTC were, also suggests a functional difference in crossmodal plasticity between primary and secondary auditory regions. Primary auditory regions are the first cortical relay of auditory inputs and have stronger subcortical inputs from the thalamus,^50^ while secondary regions might be more likely to be modulated by top-down influences, potentially driving plastic reorganization in different directions. Further studies focusing on finer-grain mapping of crossmodal plasticity effects in the auditory cortex of deaf individuals are needed to elucidate these processes.

Task switching requires cognitive flexibility and shifting between different sets of rules.^51,52^ Shifting is considered one of the core components of executive control. It is defined as the ability to flexibly shift ‘back and forth between multiple tasks, operations or mental sets’.^53^ Shifting is also an important component of working memory tasks previously shown to recruit posterior superior temporal regions in deaf individuals (e.g. two-back working memory, visuospatial delayed recognition^4,5^). In the present study, the working memory task did not significantly activate any temporal ROIs. The working memory task used in this study requires updating of information and incremental storage, but no shifting between targets or internal representations of stimuli, as required in an n-back task. Together, these results suggest that previous working memory effects in superior temporal regions are not necessarily linked to storage, updating or control, but are more likely linked to shifting between tasks or mental states.

A change of function in the auditory cortex, specifically in the right hemisphere, could be explained by the anatomical proximity to the middle temporal lobe or to the parietal lobe, specifically the temporoparietal junction (TPJ).^7,54^ Right TPJ is a multisensory associative region involved in reorientation of attention to task-relevant information, such as contextual cues or target stimuli.^55,56^ Regions of the right middle temporal gyrus have also been shown to be involved in task switching^57^ and to encode task-set representations.^58^ In the absence of auditory inputs throughout development, the proximity to the TPJ and the middle temporal gyrus may result in changes in the microcircuitry or in the computations performed by the adjacent auditory cortices, where these regions now perform computations that allow switching between tasks.^7,54,58^ This is particularly relevant for the right hemisphere, where activity in auditory regions was more strongly linked to behavioural outcomes in the switching task in the group of deaf individuals.

Another possibility is that the recruitment of ‘auditory’ temporal regions for switching observed in deaf adults reflects vestigial functional organization present in early stages of development. Research on hearing children has found activations in bilateral occipital and superior temporal cortices during task switching,^59^ with a similar anatomical distribution to the one we find here. Our findings in deaf individuals suggest that executive processing in temporal cortices could be ‘displaced’ by persistent auditory inputs that, as the individual develops, may require more refined processing or demanding computations. Thus, an alternative view is that regions considered to be ‘sensory’ have mixed functions in infants and become more specialized in adults. These regions could follow different developmental pathways influenced by environmental sensory experience. As such, the temporal regions of hearing individuals will become progressively more specialized for sound processing, whereas, in deaf individuals, they will become more specialized for subcomponents of executive processing.

The direct relationship between behavioural outcomes and activity in reorganized cortical areas is robust evidence of the functional importance of the observed crossmodal plasticity. We found that neural activity, specifically in the right temporal ROI, predicted reaction times in the switching task in the deaf group. Specifically, higher neural switch cost was linked to higher reaction time switch cost (SwitchCost_RT_), which suggests effortful processing, as previously described in other cognitive tasks with different levels of complexity.^60,61^ It is important to highlight that there were no differences in SwitchCost_RT_ between the groups, showing that the potential reliance on different neural substrates to solve the switching task does not translate into differences in performance. In fact, significant interactions between group and condition for the switching task were only found in accuracy (SwitchCost_ACC_), which in our analysis was not predicted by neural activity, but rather, by language proficiency. Executive performance has been previously associated with language proficiency in deaf children.^47,48,62–64^ While in our study language *z*-scores predict only 20.7% of the variance in SwitchCost_ACC_ and the model was only significant at *P* < 0.05, our findings suggest that language development can have long-lasting effects on executive processing throughout the lifespan. Different theories propose that language can provide the necessary framework for higher-order (if–if–then) rules to develop and be used in a dynamic task in the most efficient way.^65,66^ These hierarchical ‘if–then’ rules could be implemented, in an automatic way, to solve the arbitrary link between stimulus and response during switching. Although participants are not required to use linguistic strategies during switching, we speculate that those who have benefited from the efficiency associated with developing such frameworks can invest fewer cognitive resources into solving this task. While the role of language in executive processing needs further investigation, it is important to consider that the timely development of a first language may boost the overall efficiency of a cognitive task, in this case switching, regardless of whether the task itself allows implementation of purely linguistic mechanisms.

It is important to take into account that all signers of BSL are bilingual to a greater or lesser degree, depending on their early language background, degrees of deafness and educational experiences.^67^ Bilinguals who frequently change languages have generally been shown to have an advantage in executive function switching tasks.^68–70^ However, it is unlikely that differences in bilingualism can explain our findings in this study. If different results between deaf and hearing participants were due to the presence or not of bilingualism, we would have expected the group of deaf individuals to have a behavioural advantage in the switching task, but that was the opposite of what we found. In addition, we have previously shown that working memory responses in the superior temporal cortex of deaf individuals cannot be explained by bilingualism.^4^ In our previous study,^4^ we compared deaf native signers to two groups of hearing individuals: (i) hearing native signers, who were bilingual in English and BSL (bimodal bilinguals); and (ii) hearing non-signers who were bilingual in English and another spoken language (unimodal bilinguals). These three populations were comparably proficient in both their languages. We found differences in the recruitment of superior temporal regions between deaf individuals and both groups of hearing participants during a working memory task, suggesting a crossmodal plasticity effect driven by different sensory experience.^4^ These effects in the superior temporal cortex could not be explained by bilingualism, because this was controlled across groups. In the present study, significant activations during the switching condition were found in the same areas where we previously found working memory activations in deaf individuals (left and right pSTC, which were defined functionally based on our previous findings; see the ‘Materials and methods’ section), suggesting that these regions are involved in specific subcomponents of executive processing as a consequence of early deafness.

In addition, as a group, deaf participants had significantly longer reaction times in all tasks. This is at odds with behavioural results from studies of deaf native signers, where the performance of this group in executive function tasks is comparable to or faster than that of typically hearing individuals (e.g. Hauser *et al*.,^46^ Marshall *et al*.,^48^ and Cardin *et al*.^4^). Native signers achieve language development milestones at the same rate as that of hearing individuals learning a spoken language, highlighting again the importance of early language access, not only for communication but also for executive processing. Deaf individuals also have faster reaction times in studies of visual reactivity,^21,71^ suggesting critical differences in performance between purely perceptual tasks, and those which weigh more strongly on executive demands, where language experience and early language acquisition could have a longer-lasting effect throughout the lifespan.

In conclusion, we show that components of executive processing, such as switching, can be influenced by early sensory experience. Our results suggest that, in the absence of auditory inputs, superior temporal regions can take on functions other than sensory processing. This could be either by preserving a function these areas performed early in childhood or by taking on new functions driven by influences from top-down projections from frontoparietal areas or adjacent temporal and parietal regions.

## Supplementary Material

awac205_Supplementary_Data

## Abbreviations

BSL: British Sign Language
BSLGJT: BSL grammaticality judgement task
EGJT: English grammaticality judgement task
HEF: higher executive function
HG: Heschl’s gyrus
LEF: lower executive function
pSTC: posterior superior temporal cortex
PT: planum temporale
ROI: region of interest
