# Supplementary material for: Sensory experience modulates the reorganization of auditory regions for executive processing
Source: Brain. 2022 Jun 2;145(10):3698–710. doi: 10.1093/brain/awac205 (PMC9586534; doi:10.1093/brain/awac205)
Supplement: awac205_Supplementary_Data [file awac205_supplementary_data.pdf]

Sensory experience modulates the reorganisation of auditory regions for executive processing

Manini, Vinogradova et al.

Supplementary Figures

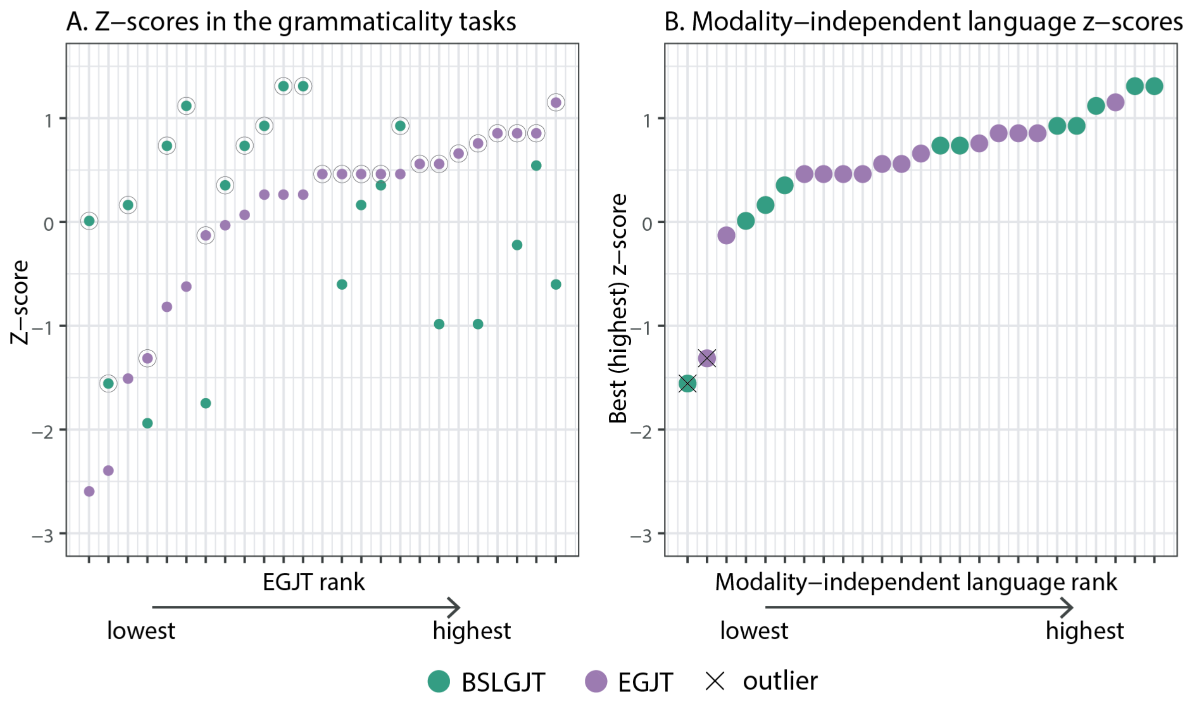

Supplementary Figure 1. Language proficiency in the deaf group. A Language z-scores in the English grammaticality judgement task (EGJT) and BSL grammaticality judgement task (BSLGJT), with participants sorted on the x-axis by their EGJT rank. Black circles indicate the z-score chosen for the combined modality-independent language score.

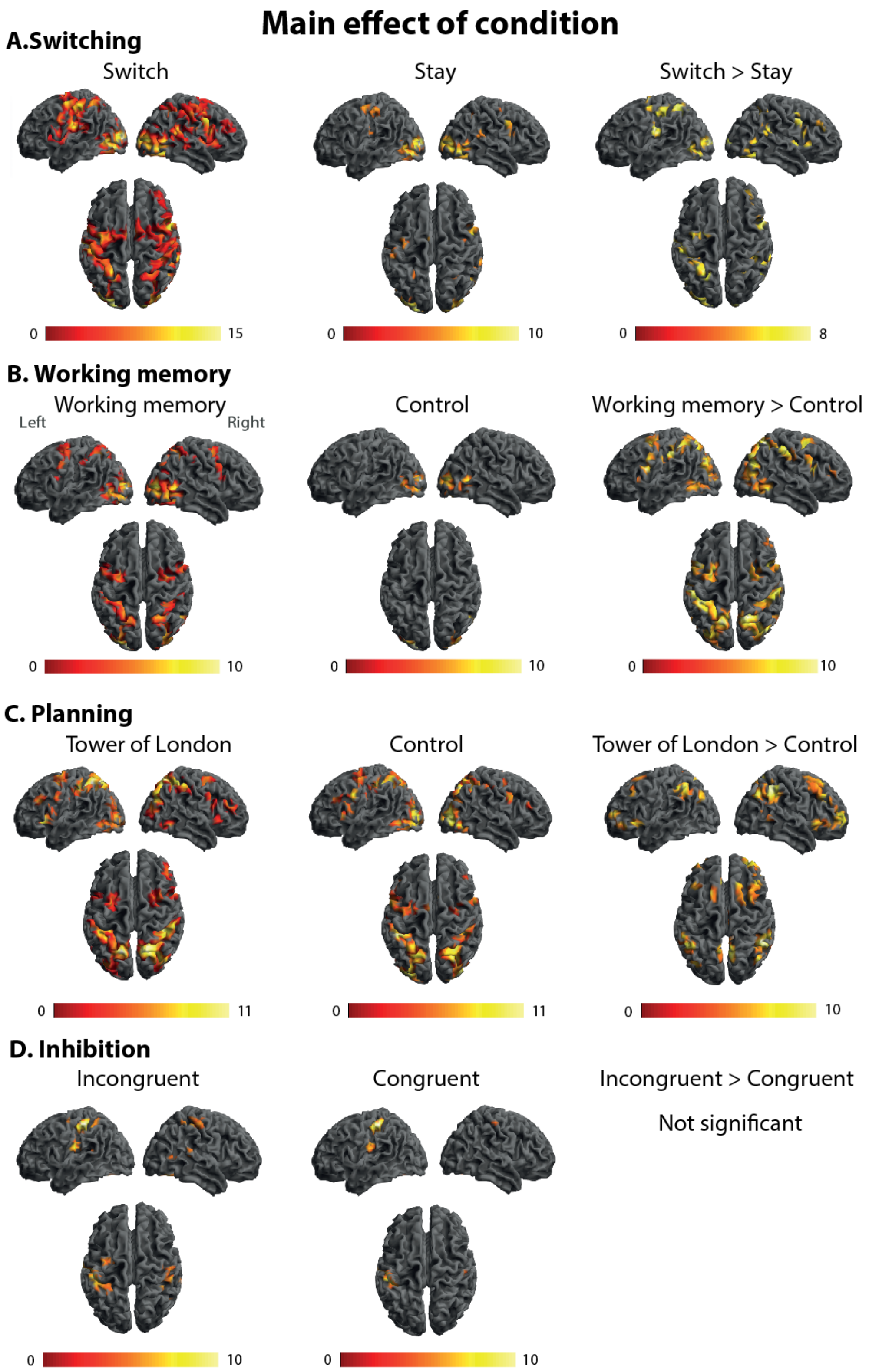

Supplementary Figure 2. Whole-brain main effects of condition in each EF task. Activations for each EF task and condition averaged across groups. All contrasts were calculated across both groups, and are displayed at $p<0.05$ (FWE-corrected). Colour bars represent z-scores. Coordinates of peak activations for the contrast [HEF > LEF] can be found here: https://osf.io/87vur/

Supplementary Tables

Supplementary Table 1. Demographics and pre-screening tests

	Age		Gender	WASI		Corsi	
	Mean (range)	SD		Mean	SD	Mean	SD
**Full sample** hearing N=20	37.50 (18-66)	16.85	15f/5m	57.47	8.02	5.4	1.1
**Full sample** deaf N=25	41.68 (19-66)	14.38	16f/9m	59.68	8.51	5.3	0.78
**Switching** hearing N=20	37.50 (18-66)	16.85	15f/5m	57.47	8.02	5.4	1.1
**Switching** deaf N=23	40.3 (19-63)	13.93	14f/9m	59.87	8.76	5.39	0.71
**WM** hearing N=19	38.47 (18-66)	16.72	14f/5m	57.83	8.1	5.47	1.08
**WM** deaf N=24	41.38 (19-66)	14.6	15f/9m	59.96	8.58	5.38	0.70
**Planning** hearing N=19	36.95 (18-66)	17.13	14f/5m	57.56	8.25	5.44	1.11
**Planning** deaf N=21	40.81 (19-63)	13.65	13f/8m	59.67	9.19	5.26	0.82
**Inhibition** hearing N=15	40.33 (18-66)	17.09	12f/3m	57.57	9.05	5.43	1.21
**Inhibition** deaf N=22	40.59 (19-66)	14.87	14f/8m	60.05	8.84	5.43	0.70

See Supplementary Table 4 for exclusion criteria from individual tasks. One participant in the hearing group did not complete the nonverbal IQ assessment[3] and the visuospatial working memory Corsi task[4].

Supplementary Table 2.

Between-group comparisons on demographics and pre-screening tests

	**N**	**N**	**Age**			**Gender**		**WASI**			**Corsi**		
	hearing	deaf	*df*	*t*	*p*	X^2	*p*	*df*	*t*	*p*	*df*	*t*	*p*
**Switching**	20	23	1,41	0.6	0.55	0.97	0.32	1,40	0.92	0.37	1,40	-0.01	0.99
**WM**	19	24	1,41	0.6	0.55	0.6	0.45	1,40	0.81	0.42	1,40	-0.36	0.72
**Planning**	19	21	1,38	0.79	0.43	0.63	0.43	1,37	0.75	0.46	1,37	-0.59	0.56
**Inhibition**	15	22	1,35	0.05	0.96	1.14	0.29	1,34	0.81	0.42	1,34	0.01	0.99

See Supplementary Table 4 for exclusion criteria from individual tasks.

Supplementary Table 3. Cause of deafness and language background of the deaf participants

Age	Onset of deafness	Cause of deafness	Native language	Preferred language	Other languages
19	0	Genetic	BSL	BSL	
21	< 3 years old	Genetic	BSL	BSL	
22	0	Genetic	English, BSL	English	
22	0	Unknown	BSL	BSL	
23	0	Genetic	BSL	BSL	ASL
27	0	Genetic	English	English	BSL
29	0	Genetic	English	English	
30	< 3 years old	Genetic	Auslan	Auslan	BSL, ASL
30	0	Unknown	English	English	BSL
40	0	Unknown	English	English	BSL
43	0	Genetic	BSL	BSL	
44	0	Mother had infection	Gesture/home sign	BSL	
45	0	Mother had rubella	English	English	BSL
47	0	Unknown	English	BSL	
48	0	Genetic	English	BSL	
49	< 3 years old	Meningitis	Gesture or home sign, BSL	BSL	
49	0	Genetic	English	English	
50	< 3 years old	Genetic	English	BSL	
51	0	Genetic	English	BSL	
52	0	Mother had rubella	English	English	
56	0	Genetic	English	BSL	
57	0	Genetic	English	English	
59	0	Genetic	BSL	BSL	
63	0	Mother had rubella	English	English	BSL
66	0	Other	English	BSL	SASL

ASL=American Sign Language, BSL=British Sign Language, SASL=South African Sign Language.
Participants' IDs have been removed to avoid identification.

Supplementary Table 4. List of participants excluded from the analysis of each task

Group	ID	Switching	Working memory	Planning	Inhibition
Hearing	3		low accuracy		not performed
Hearing	4				not performed
Hearing	6				not performed
Hearing	11				low accuracy
Hearing	13				not performed
Hearing	16			low accuracy	
Deaf	103	not performed		not performed	
Deaf	104				not performed
Deaf	106			low accuracy	not performed
Deaf	113	low accuracy	low accuracy		low accuracy
Deaf	114			movement	
Deaf	130			low accuracy	

"Low accuracy" means the participant had <55% correct answers for that task.

Supplementary Table 5.
Results from repeated-measures ANOVAs on behavioural performance

	Switching		WM		Planning		Inhibition	
	**Accuracy**							
	F (df)	*p*	*F (df)*	*p*	*F (df)*	*p*	*F (df)*	*p*
**Condition**	**28.27 (1,41)**	**<0.001**	**91.59 (1,41)**	**<0.001**	**46.88 (1,38)**	**<0.001**	**17.57 (1,35)**	**<0.001**
**Group**	**4.32 (1,41)**	**0.04**	0.03 (1,41)	0.86	0.86 (1,38)	0.36	0.24 (1,35)	0.63
**Condition × Group**	**4.98 (1,41)**	**0.03**	0.3 (1,41)	0.59	0.01 (1,38)	0.92	0.00 (1,35)	0.98
	**Reaction time**							
	F (df)	*p*	*F (df)*	*p*	*F (df)*	*p*	*F (df)*	*p*
**Condition**	**21.6 (1,41)**	**<0.001**	**199.22 (1,41)**	**<0.001**	**211.64 (1,38)**	**<0.001**	**79.2 (1,35)**	**<0.001**
**Group**	**4.5 (1,41)**	**0.04**	**8.11 (1,41)**	**0.007**	**10.96 (1,38)**	**0.002**	**4.91 (1,35)**	**0.03**
**Condition × Group**	0.03 (1,41)	0.87	0.0 (1,41)	0.97	0.06 (1,38)	0.8	0.35 (1,35)	.56

Factors in the analysis were: condition (HEF, LEF) and group (hearing, deaf). Significant effects are shown in bold.

Supplementary Table 6.
Paired samples t-tests (switch v stay) for each group and ROI in the switching task.

	**Hearing group** *df= 19*						**Deaf group** *df= 22*					
	**Left hemisphere**			**Right hemisphere**			**Left hemisphere**			**Right hemisphere**		
	t	*p*	*d*	*t*	*p*	*d*	*t*	*p*	*d*	*t*	*p*	*d*
**Heschl's gyrus**	0.41	0.68	0.10	0.35	0.73	0.08	**3.38**	**0.003**	**0.71**	1.58	0.13	0.33
**Planum temporale**	0.21	0.84	0.05	0.27	0.79	0.06	**3.55**	**0.002**	**0.74**	**5.25**	**<0.001**	**1.10**
**Posterior superior temporal cortex**	-0.22	0.82	-0.05	0.58	0.57	0.13	**3.32**	**0.003**	**0.69**	**5.31**	**<0.001**	**1.11**

Significant results are shown in bold. The following correction for multiple comparison was applied: results are considered significant when $p<0.004$ ($p<0.05/12 = 0.004$; corrected $p<0.05$).
